# Perforating invasive hydatidiform mole a catastrophe: A case report

**DOI:** 10.6026/973206300211140

**Published:** 2025-05-31

**Authors:** Suman Lakra, Sasmita Mallick, Sujata Singh

**Affiliations:** 1 Department of OBG, Government Medical College and Hospital, Sundargarh, Odisha, India; 2 Department of OBG, Dharanidhar Medical college & hospital, Keonjhar, India

**Keywords:** Fertility-preserving surgery, hemoperitoneum, invasive mole, uterine rupture

## Abstract

A hydatidiform mole, also known as molar pregnancy, is a form of gestational trophoblastic disease (GTD) characterized by abnormal trophoblastic proliferation originating from the placenta. An invasive mole, a rare but potentially life-threatening variant of GTD, is defined by the invasion of trophoblastic tissue into the myometrium and, in some cases, beyond the uterus. We present the case of a 30-year-old multiparous woman who presented with persistent vaginal bleeding and lower abdominal pain approximately one month after self-administering an oral abortifacient. Emergency surgical intervention was required in this case to control hemorrhage and stabilize the patient. However, surgical intervention may become necessary to deal with hemoperitoneum where required.

## Background:

Gestational trophoblastic disease (GTD) is a unique group of disorders that arise from abnormal proliferation of trophoblastic tissue, with hydatidiform mole representing the most common form. Complete and partial moles are its two major subtypes, with complete moles carrying a higher risk of progression to invasive disease or malignancy [1]. Although most cases of hydatidiform mole follow a benign course and respond well to evacuation, a small proportion develop into invasive hydatidiform moles, which can penetrate the uterine myometrium, invade surrounding structures or metastasize [2]. Invasive hydatidiform mole is a rare but significant complication of GTD, occurring in approximately 10-15% of patients with complete moles. The condition is characterized by persistent trophoblastic proliferation and myometrial invasion, often accompanied by elevated β-human chorionic gonadotropin (β-hCG) levels and abnormal uterine bleeding [3]. When an invasive mole perforates the uterine wall, it becomes a medical emergency, potentially leading to catastrophic outcomes such as uterine rupture, massive hemorrhage and hemodynamic instability [4]. Perforating invasive hydatidiform mole is particularly rare and its clinical presentation can be highly variable. Some patients present with nonspecific symptoms such as abnormal uterine bleeding, pelvic pain, or anemia, while others experience acute, life-threatening events, including hemoperitoneum or hypovolemic shock due to uterine rupture [5].

Reproductive function can be preserved in the majority of women with gestational trophoblastic illness [6]. Given the rarity of this condition and its potentially devastating consequences, timely diagnosis and management are essential. However, the nonspecific and overlapping clinical features of GTD with other gynecological or obstetric conditions pose a diagnostic challenge [7]. Imaging studies such as transvaginal ultrasonography and computed tomography (CT) scans, along with histopathological examination, play a critical role in establishing the diagnosis [8]. The management of perforating invasive mole requires a multidisciplinary approach involving gynecologists, oncologists, radiologists and, in severe cases, critical care specialists. Treatment typically involves uterine evacuation, systemic chemotherapy to control trophoblastic proliferation and, in cases of significant hemorrhage or uterine rupture, surgical intervention such as hysterectomy [9]. Post-treatment surveillance of β-hCG levels is critical to ensure complete remission and to monitor for recurrence [10]. This case report presents a rare instance of perforating invasive hydatidiform mole, shedding light on the clinical course, diagnostic challenges and management strategies for this life-threatening condition. By documenting this case, we aim to contribute to the growing understanding of this rare pathology, underscore the importance of early recognition, and highlight the need for timely, coordinated care to prevent fatal outcomes.

## Case report:

We present a case of perforating invasive mole in a 30-year-old woman who developed shock and hemoperitoneum. An invasive hydatidiform mole is considered a borderline or biologically uncertain tumor by the World Health Organization (WHO). Clinically, it is classified as a malignant tumor and it can sometimes present as part of gestational trophoblastic neoplasia (GTN), which includes conditions such as choriocarcinoma. Due to its distinct biological behavior, GTN is unique in that it is the first solid tumor that can be cured with chemotherapy.The patient, a 30-year-old woman (Gravida 5, Para 3, Living 3, Abortion 1), had three previous normal deliveries and one spontaneous abortion at 12 weeks. She was referred to SCBMCH, Cuttack from a nearby district for management of vaginal bleeding, hypovolemic shock, and severe anemia. She had a history of self-administered oral abortifacient one month ago, at approximately 2 months of gestation. Following this, she did not experience vaginal bleeding or passage of any mass. The patient now presented with vaginal bleeding and lower abdominal pain lasting for 1 day.On evaluation, the patient's urine pregnancy test (UPT) and paracentesis were both positive. She had no significant past medical history. Her examination revealed cold and clammy extremities, with a pulse rate of 168 beats per minute, blood pressure of 90 mmHg (systolic) and a respiratory rate of 24 breaths per minute. The abdomen was tense, tender, with guarding and rigidity. The uterus was approximately 20 weeks in size. On vaginal examination, the cervix was closed, the bilateral fornices were full, and there was no active bleeding or cervical motion tenderness. Unfortunately, the patient did not have prior blood tests or ultrasound records available. An ultrasound showed bulky uterus with anechoic cysts of varying sizes, with hyperechoic material protruding through the myometrium and free fluid, which raised concern for hemoperitoneum, resulting in a somewhat unclear clinical picture. Her β-hCG level was significantly elevated at 88,000 IU/L, confirming the diagnosis of a molar pregnancy. Given her condition, the patient was taken for emergency exploratory laparotomy after obtaining her consent.An obstetric hysterectomy with bilateral salpingo-oophorectomy was performed to manage a perforation in the posterior uterine wall caused by the invasive molar pregnancy. Intra-operatively, both ovaries were found to be cystic. The patient received a total of five units of packed red blood cells (PRBCs) and achieved hemodynamic stability in the postoperative period.

On the second postoperative day, further examinations were conducted:

Chest X-ray: No abnormalities.

Abdominal ultrasound: No free fluid or abnormalities detected.

MRI brain: No evidence of intracranial metastasis.

Serum TSH: 3.42 mIU/L within the normal range

Her WHO risk score was assessed as 6. She was carefully followed up for six months, during which her β-hCG levels gradually declined and eventually fell below 1 IU/L.

## Discussion:

Invasive hydatidiform mole is a rare but severe complication of gestational trophoblastic disease (GTD), characterized by the aggressive invasion of trophoblastic tissue into the myometrium, with the potential for uterine perforation and systemic complications [3]. This case highlights the catastrophic presentation of perforating invasive hydatidiform mole, which remains a life-threatening condition if not promptly diagnosed and managed. A 30-year-old multiparous woman, presented with hypovolemic shock, severe anemia and abdominal pain following the use of an oral abortifacient. The history of persistent abnormal uterine bleeding and the absence of expulsion of molar tissue after the abortifacient use were significant clinical red flags, compounded by the patient's delayed presentation. The markedly elevated β-hCG levels 88,000 mIU/mL provided a strong diagnostic clue for GTD, while the clinical findings of abdominal guarding, rigidity and uterine enlargement raised suspicion for uterine rupture or invasive disease. The absence of prior imaging studies or blood investigations posed a diagnostic challenge, necessitating urgent surgical intervention. Emergency exploratory laparotomy confirmed a perforating molar pregnancy with posterior uterine wall rupture, requiring a lifesaving obstetric hysterectomy [11]. The histopathological diagnosis of invasive hydatidiform mole validated the clinical and intraoperative findings [12]. Invasive mole is often misdiagnosed due to its nonspecific presentation which can mimic other obstetric or gynecological conditions such as incomplete abortion, ectopic pregnancy, or uterine leiomyoma. The lack of imaging or blood investigations in this case further complicated preoperative diagnosis [13]. Transvaginal ultrasonography, typically the first-line imaging modality, often reveals a heterogeneous intrauterine mass with cystic spaces. In cases of uterine perforation, free fluid or hemoperitoneum may also be detected [14]. In this patient, emergency surgery was necessary due to the acute clinical presentation. β-hCG levels play a critical role in the diagnosis and management of GTD. Persistently elevated levels following evacuation of molar pregnancy are highly suggestive of invasive disease. The patient's β-hCG level of 88,000 mIU/mL was consistent with the diagnosis, guiding the clinical decision-making process. The cornerstone of invasive mole management is uterine evacuation and, in cases of severe complications such as perforation, hemorrhage, or extensive invasion, surgical intervention is warranted [15]. In this case, the uterine rupture necessitated hysterectomy, which successfully controlled the bleeding and stabilized the patient. Chemotherapy, often used for non-metastatic and metastatic GTD, was not required due to the absence of systemic spread and the patient's remission following surgery, as indicated by the decline in β-hCG levels to <1 mIU/mL over six months [16]. A class of aggressive fertilisation illnesses known as gestational trophoblastic neoplasia is defined by the invasion of the uterine endometrial and myometrial layers by malignant trophoblastic cells [17]. Long-term follow-up is essential in GTD management to ensure complete remission and to monitor for recurrence. Serial β-hCG measurements remain the gold standard for follow-up, as persistent or rising levels may indicate residual trophoblastic disease or malignant transformation. The patient's six-month follow-up with undetectable β-hCG levels confirmed successful management of the invasive mole. Additionally, imaging studies, including chest X-ray and MRI, ruled out metastasis, further supporting the positive prognosis.

[1] Early recognition and diagnosis: A high index of suspicion is crucial in women presenting with abnormal uterine bleeding, elevated β-hCG levels, and a history of incomplete abortion or failed abortifacient use.

[2] Urgency of intervention: Delayed diagnosis can result in catastrophic complications such as uterine perforation and hypovolemic , as seen in this patient.

[3] Role of multidisciplinary care: Successful management often requires collaboration between gynecologists, oncologists, radiologists, and critical care specialists, particularly in acute cases.

[4] Importance of follow-up: Regular β-hCG monitoring is vital to ensure remission and detect early signs of recurrence or malignant transformation.

## Conclusion:

This case illustrates the potentially life-threatening nature of an invasive hydatidiform mole complicated by uterine perforation and hemoperitoneum. While suction evacuation is the standard initial treatment for molar pregnancy, surgical intervention may be necessary in unstable patients or when complications arise. Close monitoring with serial serum β-hCG measurements is essential for early detection of persistent trophoblastic disease. In some cases, hysterectomy with ovarian preservation remains a valuable treatment option.
